# 

**Published:** 1896-03

**Authors:** 


					﻿Vol. XVI.
MARCH, 1896.
NO. 3.
THE
HOMEOPATHIC pHY^lClAp,
A Monthly Journal of Homoeopathic Materia Medica
and Clinical Medicine.
“He it a freeman whom truth makes free, and all are slaves betide."
"Seek the Truth: come whence it may, cost what it will."
EDITED AND PVBLMHED BY v
WALTER M. JAMES, M. D.
PHILADELPHIA :
USTo. 1231 LOCUST STREET.
x.oit£>O2? ;
ALFRED HEATH & CO., 8ole Agents fob Great Britain,
114 Ebury Street, Eaton Square.
‘Yearly Subscription, $2.50, invariably in advance. Sinyle numbers, 25 ets.
Entered at the Philadelphia Poit-Offiee aa Second-Claaa Mall Matter.
				

